# Musculoskeletal pain in 13-year-old children: the generation R study

**DOI:** 10.1097/j.pain.0000000000003182

**Published:** 2024-02-07

**Authors:** Guido J. van Leeuwen, Marleen M. van den Heuvel, Patrick J. E. Bindels, Sita M. A. Bierma-Zeinstra, Marienke van Middelkoop

**Affiliations:** Departments of a General Practice and; b Orthopedics and Sports Medicine, Erasmus MC University Medical Center Rotterdam, Rotterdam, the Netherlands

**Keywords:** Musculoskeletal pain, Children, Adolescents, Generation R, Physical factors, Psychosocial factors

## Abstract

Musculoskeletal (MSK) pain is a common reason for consultation in general practice and frequently reported in children and adolescents. This study examined the prevalence of MSK pain in 13-year-old children and assessed associations with physical and psychosocial factors. Data from the Generation R Study, a population-based birth cohort, was used. Prevalence and characteristics of MSK pain were assessed, using a pain mannequin, at 13 years of age (N = 3062). Demographics and data on physical activity, sedentary behaviors, previous reported MSK pain, and behavioral problems were extracted from questionnaires. The body mass index (BMI) SD-score was calculated from objectively measured weight and height. A prevalence of 23.3% was found for MSK pain in children of which 87.2% persisted for more than 3 months (ie, chronic), 45.5% experienced pain daily. More physically active children and children with a higher BMI reported MSK pain more frequently compared with non-MSK pain and no pain. The knee was the most often reported location. Children with MSK pain were more likely to have reported MSK pain at 6 years. Multivariable analyses showed significant associations for male sex (OR 0.74, 95% CI 0.56-0.98), high maternal educational (OR 0.69, 95% CI 0.49-0.96), higher BMI (OR 1.19, 95% CI 1.05-1.35), being physically active (OR 1.41, 95% CI 1.03-1.91), and behavioral problems (OR 1.85, 95% CI 1.33-2.59) with the presence of MSK pain. The chronic nature of MSK pain in combination with the relatively high prevalence of MSK pain in this study shows that MSK pain is already an important problem at a young age.

## Abstract 2

In 13-year-old children, musculoskeletal pain has a relatively high prevalence and is a chronic and often daily problem.

## 1. Introduction

Musculoskeletal (MSK) pain is one of the most common health problems in children and adolescents and is associated with significant individual, social, and financial burden worldwide and is one of the leading causes of years lived with disability.^12^ Musculoskeletal problems can already have extensive influence on their normal daily routine of school, social, and sports participation.^13,17,37,38^ Research has reported prevalence rates of MSK pain in children aged 9 to 18 years ranging from 4% to 40%^24^ and that 1 in 3 adolescents have MSK pain monthly.^22^ Knee, back, and neck pain are the most common sites of musculoskeletal pain.^24^

Many factors, both physical and psychosocial, are suggested to influence the presence of MSK pain in children. Physical factors as skeletal growth and maturation are believed to be main reasons for MSK problems to arise in childhood and adolescence.^15^ Nontraumatic knee complaints, such as Osgood–Schlatter disease (OSD) and patellofemoral pain (PFP), are typical knee problems that emerge during puberty.^7,23,39^ Furthermore, research suggests a possible association between MSK problems during childhood and development and the chronic character of MSK problems in adults.^3,40^ A history of MSK pain at 11-year olds is a very strong risk factor for MSK pain at age 14 years.^34^ Some studies have investigated possible associations between various physical, joint loading factors and the development of MSK problems in children and adolescents. For example, being overweight is significantly associated with the presence of MSK pain in childhood,^31^ and taller children seem more likely to develop MSK problems than shorter peers,^18^ possibly because of increased joint loading and dysfunction.^27^ Some research indicates a relation between another joint loading factor as physical activity and exercise and the onset of low back pain and spinal pain in children.^10,18^ However, in another study in 14-year-old children, no associations were found between physical activity and MSK pain.^34^ Furthermore some research has shown a possible association between sedentary behavior and MSK pain in children and adolescents,^21^ whereas other studies did not find this association.^18,34^ The association between psychosocial factors and MSK pain in children has been studied, with associations found between negative emotional symptoms,^18^ worrying, sleeping problems,^34^ and externalizing problems.^16^ These results are similar to what has been found in adults.^30,47^ Nevertheless, for most of the aforementioned factors, there is an absence of high-quality prospective studies and thus the evidence for their proposed potential as risk factors remains conflicting. In the Generation R study,^25^ it was found that MSK pain at the age of 36 months and behavioral problems are independently associated with the presence of MSK pain in 6-year-old children.^45^ Currently, similar studies in 13-year-old children have not been performed yet, although it is known that MSK complaints often develop during adolescence.^33,48^ Therefore, the aim of this study is to describe the prevalence and characteristics of MSK pain in 13-year-old children in a large population-based birth cohort and identify physical and psychosocial factors associated with MSK pain.

## 2. Methods

### 2.1. Study population

This study was performed within in the Generation R Study, a population-based prospective cohort study, focusing on growth, development, and health from fetal life until young adulthood. Eligible participants were women with an expected delivery date between April 2002 and January 2006, living in Rotterdam, the Netherlands. At the start of the study, 9749 children of the pregnant mothers were included in the cohort. At regular time points during infancy, childhood, and puberty, questionnaires were send out, and physical examination was performed. Detailed information on the Generation R study cohort has been described elsewhere.^25^ The Medial Ethical Committee of the Erasmus Medical Center, Rotterdam, approved the study, and written informed consent was obtained from the parents of all participants.

### 2.2. Procedures

For this study, data from the follow-up phases at the age of 6 and 13 years were used. In total, 7968 children still participated in the study at the age of 13 years, 82% of the original cohort. Data were derived from physical examinations at the research center and from self-reported and parent-reported questionnaires. Questions on pain were asked with a self-reported questionnaire during a second visit at the research center, which was arranged for the purpose of magnetic resonance imaging (MRI). Children who took part in the regular first visit regular measurements at 13 years received an invitation to participate in the MRI study. Children who did not participate in the first visit or declined to participate in the MRI study were excluded for this study purpose. This resulted in a final study sample of 3062 children with data on the presence of pain and pain locations. Additional questions on pain characteristics were added to the questionnaire in a later stage after delayed ethical approval procedures and are therefore only available in 325 children. Reporting in this study is in accordance with STROBE reporting guidelines.

### 2.3. Measurements—questionnaires

#### 2.3.1. Demographics

Information on child's sex was obtained from midwife and hospital records at birth. Maternal and paternal educational level was categorized to high (higher education, master's degree), intermediate (higher education, bachelor's degree), and low (no education finished, primary school, or secondary school). Country of birth of both parents was assessed by prenatal questionnaires and, if necessary, supplemented and corrected according to follow-up questionnaires. Based on the average income in the Netherlands, the net household income was dichotomized into less or more than €1600 per month. The Tanner stage, a scale that defines physical measurements of development based on external primary and secondary sex characteristics in children,^28,29^ derived from the questionnaire was used to define puberty. Puberty was defined as Tanner stage 3 or higher.

#### 2.3.2. Musculoskeletal pain

To assess the presence of pain, children were asked whether they had pain in the past 6 weeks. If so, the location was checked on a pain mannequin^46^ with 61 possible locations, both at the front and back side of the body. In this study, MSK pain was defined as pain in neck, back, or upper and lower limbs (Supplementary Figure 1, available at http://links.lww.com/PAIN/B999). Additional questions on pain characteristics included the frequency of occurrence (constant/daily/multiple days per week/weekly/monthly/less than monthly), pain duration (number of weeks or months), onset of pain (sudden/gradual), relation to sports (yes/no), and pain intensity (numeric rating scale 0-10).

For the history of MSK pain, data from the parent-reported questionnaire at the age of 6 years, based on the pain list,^32^ was used. In that questionnaire, parents were asked whether their children had any pain in the past 3 months, defined as pain recurring or lasting for longer periods and not a result of falling or bumping. If present, they were asked to choose the pain location from a list of 9 locations. Musculoskeletal pain at the age of 6 years was defined as back pain, neck pain, or limb pain from that list.

#### 2.3.3. Physical activity and sedentary behaviors

Questionnaire data were used to evaluate physical activity and sedentary behaviors. Various physical activity behaviors were evaluated, namely, physical activity (ie, being physically active), sports participation (yes or no), and active transport to/from school. Physical activity was derived from the number of days per week with at least 1 hour of physical activity. The cutoff for a child being physical active was defined as at least 1 hour per day for at least 4 days per week (ie, more than half of the week). Active transport to/from school was defined as having at least 1 trip of walking or cycling to school per week.

Television viewing (including video/DVD) and computer use (including video games) were used to measure sedentary behaviors. The number of days and minutes per week and weekend days that children viewed television or used the computer were asked. The mean daily television viewing and computer use were calculated by dividing the sum of mean hours per day by 7. Daily television viewing was dichotomized into ≥1 hour/day vs <1 hour/day and daily computer use into ≥2 hour/day vs <2 hour/day.

#### 2.3.4. Child behavior

Among the Generation R participants The Child Behavior Checklist (CBCL) was used to assess behavioral problems.^2^ The CBCL is known for having good reliability, validity, and generalizability and is widely used to asses behavioral and emotional problems in children.^2,19^ The CBCL questionnaire consists of 99 items divided in 7 subscales: Emotionally Reactive, Anxious/Depressed, Somatic Complaints (without medical cause), Withdrawn behavior (from social contacts), Sleep Problems, Attention Problems, and Aggressive Behavior. For the analyses, the total CBCL problem score, and the sum scores on internalizing problems (Emotionally Reactive, Anxious/Depressed, Somatic Complaints, and Withdrawn scales) and externalizing problems (Attention Problems and Aggressive Behavior scales) were calculated.^2^ Besides the continuous scores, where a higher score indicates more behavioral problems, the number of children with (sub)clinical problems was calculated following the cutoff scores from a Dutch reference group. Total score, internalizing score, and externalizing score were based on the 84th percentile and somatic complaints on the 93rd percentile. These cutoff scores are the standard for defining borderline/clinical problems, which includes enough problems to be of concern (borderline ranges), and so many problems that a child clearly deviates from norms for the child's sex and age range (clinical ranges).^2,44^

### 2.4. Measurements—physical examination

#### 2.4.1. Anthropometry

Child height was measured in standing position using a Harpenden stadiometer (Holtain Limited, Crymych, United Kingdom), and weight was measured without heavy clothing and shoes using a mechanical personal scale (SECA). Height and weight were used to calculate the body mass index (BMI) (kilograms/square meter). Body mass index SD scores account for child age and sex and were calculated based on the Dutch reference growth curves, where a BMI SD score of 0 correlates with a child being on the 50th percentile.^11^ Weight status was categorized according to the cutoffs by Cole and Lobstein^5^ and dichotomized to underweight or normal weight vs overweight or obesity. Overweight was defined as being on the 90th percentile, which correlates with a BMI SD score of roughly 1.3.

### 2.5. Statistical analyses

Child characteristics and prevalence of pain were described using descriptive statistics. To analyze the differences between children with MSK pain, children with pain at other sites (ie, “other pain”), and children without pain (ie, “no pain”), the χ^2^ and analysis of variance (normally distributed) were used for categorical variables and Mann–Whitney U (2 groups) and Kruskal–Wallis (>2 groups) tests were used for continuous variables. Post hoc analyses were performed to assess which of the 3 subgroups significantly differed from each other. Furthermore, an exploratory analysis into the differences between the children who did and did not participate in the MRI study was performed. In addition, multinomial logistic regression, both univariate and multivariable, expressed in odds ratios (ORs) with 95% CIs was performed to test associations between demographics, physical, and psychosocial factors with the presence of MSK pain compared with children with no pain. The regression analyses were performed in a sample of 1612 children with complete data on all predictors. Redundant variables were removed from the multivariable regression model in case of collinearity, assessed by correlation coefficients with a cutoff of 0.7. All analyses were conducted with nonimputed data. Depending on the number of missing values in the predictor, the sample size for the univariate analyses differed per analysis (ranging from 0% missing data for sex and age to 29.6% for active transport to/from school). SPSS software (IBM Corp. Released 2016. IBM SPSS Statistics for Windows, Version 25.0. Armonk, NY: IBMCorp) was used for all analyses, and the level of statistical significance was set at *P* < 0.05.

## 3. Results

### 3.1. Participants characteristics

Almost half (49.7%) of the 3062 included children were boys (Table 1). The children had a median age of 13.8 years (interquartile range 13.6-14.4 years), with a BMI SD score of 0.44 (SD 1.19) and Dutch born parents in 61.8%. In 38.7% of the children, the maternal educational level was low. Compared with the excluded participants, participants in this study (N = 3062) were significantly less often boys, had an older age, more often had a Dutch ethnicity, and had a higher maternal education (Supplementary Table 1, available at http://links.lww.com/PAIN/B999).

**Table 1:** Participant characteristics.

	Total (n = 3062)	MSK pain (n = 575)	Other pain (n = 139)	No pain (n = 2348)	*P*
Demographics					
Sex					**0.034**
Girl	1594 (52.1%)	321 (55.8%)^a^	78 (56.1%)	1195 (50.9%)^a^	
Boy	1468 (47.9)	254 (44.2)^a^	61 (43.9)	1153 (49.1)^a^	
Age, y	13.81 (13.58-14.36)	13.84 (13.58-14.48)	13.93 (13.65-14.57)^a^	13.80 (13.58-14.33)^a^	**0.048**
Birth country of parents					
Dutch	1853 (61.8)	339 (60.0)	68 (50.0)^a^	1446 (62.9)^a^	**0.019**
Other Western	270 (9.0)	55 (9.7)	12 (8.8)	203 (8.8)	
Non-Western	877 (29.2)	1771 (30.3)	56 (41.2)^a^	650 (28.3)^a^	
Maternal educational level					
High	852 (32.1)	138 (27.8)^a^	23 (19.7)^b^	691 (33.8)^a,b^	**0.001**
Intermediate	778 (29.3)	147 (29.6)	34 (29.1)	597 (29.2)	
Low	1028 (38.7)	212 (42.7)^a^	60 (51.3)^b^	756 (37.0)^a,b^	
Paternal educational level					
High	925 (38.0)	146 (33.0)^a^	30 (29.1)^b^	749 (39.7)^a,b^	**0.010**
Intermediate	583 (24.0)	105 (23.7)	25 (24.3)	453 (24.0)	
Low	925 (38.0)	192 (43.3)^a^	48 (46.6)^b^	685 (36.3)^a,b^	
Household income, <€1600/mo	339 (13.4)	73 (15.7)	23 (20.0)^a^	243 (12.4)^a^	**0.019**
Single parenthood, yes	337 (12.8)	77 (15.6)^a^	18 (15.3)	242 (11.9)^a^	0.066
Physical factors					
BMI, SD score	0.44 (1.19)	0.58 (1.18)^a^	0.57 (1.23)	0.39 (1.19)^a^	**0.001**
Overweight, yes	486 (15.9)	115 (20.0)^a^	31 (22.3)^b^	340 (14.5)^a,b^	**0.001**
Height, SD score	0.01 (1.01)	0.05 (0.99)^a^	−0.20 (0.98)^a,b^	0.01 (1.02)^b^	**0.033**
Puberty					
Tanner stage ≥3, yes	1479 (61.3)	286 (64.3)	63 (63.6)	1130 (60.4)	0.289
Physical activity behaviors					
Physical activity					
Minimum 1 h/d ≥ 4 d/wk, yes	1487 (66.8)	291 (72.0)^a,b^	52 (55.3)^a,c^	1144 (66.2)^b,c^	**0.004**
Sports participation, yes	2160 (84.8)	417 (88.3)^a,b^	86 (80.4)^a^	1657 (84.2)^b^	**0.035**
Active transport, ≥ 1 trip/wk	1909 (88.6)	342 (87.0)	72 (84.7)	1495 (89.1)	0.254
Sedentary behaviors					
Television viewing, h/d	0.79 (0.50-1.79)	0.79 (0.50-1.79)	1.21 (0.50-1.79)	0.79 (0.50-1.79)	0.118
Television viewing, ≥1 h/d	1037 (47.1)	197 (49.6)	49 (52.7)	791 (46.2)	0.251
Computer game use, h/d	3.00 (2.00-4.43)	3.29 (2.00-4.71)	3.43 (2.00-5.00)	3.00 (1.93-4.43)	0.091
Computer game use, ≥2 h/d	1631 (75.4)	314 (78.9)	70 (76.9)	1247 (74.4)	0.170
Previous MSK pain					
MSK pain at 6 y	276 (10.5)	66 (13.3)^a^	16 (14.3)	194 (9.6)^a^	**0.022**
Psychosocial factors					
Child behavioral problems (CBCL)					
Total problems, score	13.11 (6.05-26.00)	15.00 (7.78-29.37)^a^	18.58 (9.75-32.21)^b^	13.00 (6.00-24.20)^a,b^	**<0.001**
(Sub)clinical total problems, yes	459 (17.0)	111 (22.3)^a^	28 (23.7)^b^	320 (15.3)^a,b^	**<0.001**
Internalizing problems, score	4.00 (1.00-8.00)	4.00 (2.00-8.00)^a,b^	6.00 (2.82-11.00)^a,c^	4.00 (1.00-7.47)^b,c^	**<0.001**
(Sub)clinical internalizing problems, yes	492 (18.2)	101 (20.3)^a^	35 (29.7)^a,b^	356 (17.0)^b^	**0.001**
Externalizing problems, score	2.00 (0.00-6.00)	3.00 (1.00-7.00)^a^	4.00 (1.00-7.21)^b^	2.00 (0.00-6.00)^a,b^	**<0.001**
(Sub)clinical externalizing problems, yes	416 (15.4)	95 (19.1)^a^	26 (22.4)^b^	295 (14.2)^a,b^	**0.002**
Somatic problems, score	1.00 (0.00-3.00)	1.00 (0.00-3.00)^a,b^	2.00 (1.00-4.00)^a,c^	1.00 (0.00-2.86)^b,c^	**<0.001**
(Sub)clinical somatic problems, yes	308 (11.4)	74 (14.9)^a^	24 (20.7)^b^	210 (10.1)^a,b^	**<0.001**

Values presented as number (%) for categorical factors, or median (interquartile range) or mean (SD) for continuous factors. Bold values represent statistically significant *P* values (*P* < 0.05).

This table is based on nonimputed data. Missing values were 0 for sex, 0 for age, 62 (2.0%) for ethnicity, 404 (13.2%) for maternal educational level, 629 (20.5%) for paternal educational level, 530 (17.3%) for household income, 422 (13.8%) for single parenthood, 2 (0.1%) for BMI, overweight and height, 648 (21.2%) for puberty, 835 (27.3%) for physical activity, 516 (16.9%) for sports participation, 907 (29,6%) for active transport, 859 (28.1%) for television viewing, 898 (29.3%) for computer use, 426 (13.9%) for MSK pain at 6 years, 357 (11.7%) for the Child Behavior Checklist (CBCL) total score, 355 (11.6%) for the CBCL internalizing problems score, 365 (11.9%) for the CBCL externalizing problems score, and 366 (12.0%) for the CBCL somatic complaints score.

^a,b,c^Subgroups that significantly differ from each other, based on post hoc analyses.

### 3.2. Reporting of pain

In the previous 6 weeks, 714 children (23.3%) reported that they experienced any type of pain and 575 children reported MSK pain (18.8%). Of the sample of children that reported MSK pain, lower limb pain was most often reported (75.5%), followed by back pain (17.2%) and upper limb pain (16.0%). Knee pain was the most reported location of MSK pain (43.7%) followed by ankle/foot pain (26.6%). Girls reported more back pain than boys (21.2% vs 12.2%), whereas lower limb pain was reported more in boys than in girls (81.1% vs 71.0%) (Table 2).

**Table 2:** Frequencies of musculoskeletal pain locations within the group of children with musculoskeletal pain and differences between boys and girls.

	Total (n = 575)	Boys (n = 254)	Girls (n = 321)	*P*
Back pain	99 (17.2)	31 (12.2)	68 (21.2)	**0.005**
Neck pain	16 (2.8)	5 (2.0)	11 (3.4)	0.291
Upper limb pain	92 (16.0)	36 (14.2)	56 (17.4)	0.288
Shoulder	36 (6.3)	13 (5.1)	23 (7.2)	0.314
Elbow	11 (1.9)	7 (2.8)	4 (1.2)	0.189
Wrist/hand	45 (7.8)	17 (6.7)	28 (8.7)	0.368
Lower limb pain	434 (75.5)	206 (81.1)	228 (71.0)	**0.005**
Hip	35 (6.1)	14 (5.5)	21 (6.5)	0.608
Knee	251 (43.7)	110 (43.3)	141 (43.9)	0.882
Ankle/foot	153 (26.6)	74 (29.1)	79 (24.6)	0.223

Values presented as number (%).

Bold indicates significant *P* values.

Most children experienced pain at only 1 pain location (83.7%), and almost half (45.8%) of the children experienced pain daily. Moreover, more than half of the children indicated that the MSK pain was related to sports (60.2%). In nearly 10% of the children with MSK pain, the pain was also experienced at other sites (8.9%), most often in the abdomen (4.5%) or the head (4.3%). Girls more frequently had MSK pain in multiple locations than boys (17.6% vs 6.0%, respectively) and reported more pain at other sites, particularly the abdomen, than boys (12.1% vs 4.7%, respectively). In boys, the pain was more often related to sports than in girls (70.0% vs 53.0% resp). Musculoskeletal pain was classified as chronic, ie, the pain existed for more than 3 months, in 87.2% of the children with pain (Table 3).

**Table 3:** Pain characteristics in musculoskeletal pain group.

	Total (n = 575)	Boys (n = 254)	Girls (n = 321)	*P*
No. of MSK pain locations				**<0.001**
1	447 (83.7)	209 (89.7)	238 (79.1)	
2	67 (12.5)	14 (6.0)	53 (17.6)	
>2	20 (3.7)	10 (4.3)	10 (3.3)	
Frequency of occurrence				0.630
<1 x/mo	30 (11.5)	13 (11.8)	17 (11.3)	
≥1 x/mo, <1 x/wk	6 (2.3)	1 (0.9)	5 (3.3)	
≥1 x/wk	105 (40.4)	46 (41.8)	59 (39.3)	
Every day	119 (45.8)	50 (45.5)	69 (46.0)	
Pain intensity, NRS	5.75 (1.60)	5.81 (1.50)	5.70 (1.67)	0.587
Pain duration, > 3 mo	157 (87.2)	57 (82.6)	100 (90.1)	0.144
Pain onset				0.467
Sudden	162 (62.5)	66 (60.0)	96 (64.4)	
Gradual	97 (37.5)	44 (40.0)	53 (35.6)	
Pain related to sports, yes	157 (60.2)	77 (70.0)	80 (53.0)	**0.006**
Pain at other sites				
Yes	51 (8.9)	12 (4.7)	39 (12.1)	**0.002**
Head	25 (4.3)	7 (2.8)	18 (5.6)	0.096
Chest	9 (1.6)	2 (0.8)	7 (2.2)	0.181
Abdomen	26 (4.5)	5 (2.0)	21 (6.5)	**0.009**

Values presented as number (%) for categorical factors, or mean (SD) for continuous factors.

This table is based on nonimputed data. Missing values were 41 (7.1%) for locations of pain, 315 (54.8%) for frequency of occurrence, 315 (54.8%) for pain intensity, 395 (68.7%) for pain duration, 316 (55.0%) for pain onset, 314 (54.6%) for pain related to sports, and 0 for pain at other sites.

Bold indicates significant *P* values.

NRS, numeric rating scale.

### 3.3. Differences between children with musculoskeletal pain, pain at other sites, or without pain

In Table 1, differences in demographics, physical factors, and psychosocial factors between the groups of children with MSK pain, pain at other sites, and without pain are presented. There were less parents with a high educational level in the MSK pain and other pain groups compared with the no pain group (maternal educational level: 27.8%, 19.7%, and 33.8% and paternal educational level: 33.0%, 29.1%, and 39.7% respectively).

A higher BMI SD score (0.58 [SD 1.18] vs 0.39 [SD 1.19], *P* = 0.001) and more children with overweight (20.0% vs 14.5%, *P* = 0.001) were seen in the MSK pain group compared with the no pain group. Regarding physical activity behaviors, the MSK pain group had a higher amount of physical activity compared with the other and no pain groups (72.0%, 55.3%, and 66.2%, respectively) and participated more frequently in sport (88.3%, 80.4%, and 84.2%, respectively). However, there were no differences between the groups in sedentary behaviors. Children with MSK pain at 13 years more often had MSK pain at 6 years than in those without pain (13.3% vs 9.6%).

Significant differences between the 3 pain groups were found for all CBCL (sub)scores. In general, the children with MSK or other pain had more behavioral problems compared with children with no pain.

### 3.4. Multivariable regression analyses on the presence of musculoskeletal pain in complete cases

In the group of 1612 children with complete data on all predictors, univariate analyses showed significant associations with the presence of MSK pain compared with no pain for high maternal educational level, BMI, and behavioral problems. In multivariable analyses, significant negative associations for the prevalence of MSK pain were seen for male sex (OR 0.74, 95% CI 0.56-0.98) and higher maternal education (OR 0.69, 95% CI 0.49-0.96), although significant positive associations were seen for BMI (OR 1.19, 95% CI 1.05-1.35), being physically active (OR 1.41, 95% CI 1.03-1.91), and behavioral problems (OR 1.85, 95% CI 1.33-2.59) (Table 4).

**Table 4:** Logistic regression analysis for musculoskeletal pain compared with no pain in complete cases (n = 1612).

	Univariate analyses, OR (95% CI)	Multivariable analyses, OR (95% CI)
Demographics		
Sex, boy	0.79 (0.61-1.02)	**0.74 (0.56-0.98)**
Age, y	1.05 (0.85-1.30)	1.03 (0.83-1.29)
Ethnicity		
Dutch	Reference	Reference
Other Western	1.16 (0.78-1.76)	1.20 (0.78-1.85)
Non-Western	1.05 (0.74-1.48)	1.00 (0.69-1.45)
Maternal educational level		
High	**0.68 (0.50-0.93)**	**0.69 (0.49-0.96)**
Intermediate	0.87 (0.64-1.19)	0.86 (0.62-1.19)
Low	Reference	Reference
Physical factors		
BMI, SD score	**1.21 (1.07-1.36)**	**1.19 (1.05-1.35)**
Height, SD score	1.01 (0.89-1.15)	0.95 (0.83-1.09)
Puberty, yes	1.22 (0.94-1.59)	1.04 (0.77-1.41)
Physical activity, minimum 1 h/d ≥ 4 d/wk, yes	1.30 (0.97-1.74)	**1.41 (1.03-1.91)**
Sports participation, yes	1.32 (0.86-2.04)	1.56 (0.98-2.49)
Computer game use, ≥2 h/d	1.18 (0.87-1.60)	1.24 (0.91-1.69)
MSK pain at 6 y, yes	1.46 (1.00-2.14)	1.42 (0.96-2.10)
Psychosocial factors		
CBCL—(Sub)clinical total problems, yes	**1.82 (1.31-2.51)**	**1.85 (1.33-2.59)**

Odds ratios (OR) and 95% confidence intervals (CIs) resulting from multinomial logistic regression, including 1612 participants (nonimputed data). Bold values represent statistically significant ORs (*P* < 0.05).

## 4. Discussion

In this study, a 6-week prevalence of 18.8% was found for MSK pain in 13-year-old children, of which almost 90% was chronic and almost half of the children experienced pain daily. The most reported location of MSK pain was the lower limbs with boys reporting more lower limb pain than girls, whereas girls reported more back pain than boys. Children who previously reported MSK pain at 6 years reported MSK pain more frequently compared with no pain. Multivariable analysis showed significant associations with the presence of MSK pain in girls, low maternal educational level, a higher BMI, higher physical activity level, and presence of behavioral problems.

This study reported a 23% 6-week prevalence for pain, which is lower than the reported prevalence rates of 40% to 60% in previous research performed in children and adolescents.^14,24,32,43^ Comparing the prevalence in this study with the prevalence in 6-year-old children, the prevalence of MSK pain rose from 10% to almost 19%.^45^ This increase is likely due to physical changes, growth spurts, increased skeletal maturation, and changes in psychosocial factors at this age.^6,15^ Moreover, the increased prevalence could be due to more detailed pain questions in the questionnaire at 13 years compared with the questions at 6 years, such as a pain mannequin and specific questions about frequency, duration, and intensity. Knee pain being the most reported location of MSK pain is in line with the most research into MSK pain in adolescents.^7,43^ However, more research specifically in children with knee pain and possible associated factors is needed because current research into knee pain in children is limited and most research is performed in adults.

Almost 90% of children with MSK pain in this study, which had available data on duration, reported chronic pain. This is substantially higher than the percentage of chronic MSK pain in children with MSK pain at 6 years of age within the same study population, namely, 35.6%.^45^ This shows that aside from the rising prevalence, MSK pain becomes more chronic with increasing age. Only 10% of chronic MSK pain children also reported MSK pain at 6 years old. This seems to imply that chronic MSK pain most children during puberty is not related to the presence of MSK pain at a younger age. Because the knee was the most frequently reported location, a possible explanation for chronic MSK pain could be specific MSK complaints that are known to arise during puberty and persist long term such as nontraumatic knee complaints as PFP or OSD.^7,23,26^ Another explanation for chronic MSK pain could be back pain, which approximately 1 in 5 children in this study reported, and previous research has shown that back pain often also is a chronic complaint in children and adolescents.^1,8^ However, in this study, no differences were seen between MSK pain location and the amount of chronic pain (data not presented). The chronic nature of MSK pain in combination with the relatively high prevalence of MSK pain in this study and known burden of disease^12^ show that MSK pain is an important problem that already warrants attention starting at puberty.

In concordance with the literature,^31^ a higher BMI SD score and more children with overweight were seen in children with MSK pain vs children without pain. It is known that being overweight or obese can impact joint health and cause joint dysfunction, which leads to more ankle, foot, and knee problems than being normal weight.^27^ It is believed that joint dysfunction in children with overweight or obesity is caused by increased joint loading resulting in structural changes as malalignment.^4,9^ Thus, more attention should be given to children with overweight and MSK complaints because this could play an important role in the treatment of MSK pain and prevent future problems.

Physically active children seem to experience more MSK pain. This finding may be due to injuries that occur during sports participation. However, at this moment, there is no clear evidence from the literature for the relation between MSK pain and physical activity level in children, as both positive and negative associations have been reported.^41^ Furthermore, details about the character and intensity of the physical activity and precise origins of pain were not available in this study, although these factors could certainly play a role in the development and presence of MSK pain. These factors should be included in future research to get a better insight in the relation between physical activity level and MSK pain. Although evidence on the association between physical activity and knee pain is inconclusive due to heterogeneity in study populations^14,32,36,43^ and absence of high-quality prospective studies, there should be more awareness for children who are physically active because of the possible higher risk on MSK pain, maybe due to injury or overloading. However, it is also not desirable for children to refrain from physical activity because of the aforementioned negative effect of sedentary behavior.^21^ Attention should be paid to both preventing injury or overloading due to physical activity and to the possible long-term nature and impact of MSK pain during rehabilitation.

Finally, the presence of MSK pain and pain in general was associated with more behavioral problems, both in univariate and multivariable analyses. This is in line with current widespread belief that psychosocial problems are an important risk factor for the onset and poorer prognosis of MSK pain in adolescents and adults.^16,30,47^ The increased presence of MSK pain could stem from increased behavioral problems due to psychosocial factors, such as depressive feelings, negative pain beliefs, cognitions, and behaviors because they are associated with the development and persistence of chronic pain.^35,42,47^ However, studies have also indicated that chronic pain leads to changes in the central nervous system and various pain modulating neural areas and can induce negative affective states, such as depression, anger, and anxiety.^49^ Because of the cross-sectional nature of this study, we are unable to determine whether more behavioral problems lead to (chronic) MSK pain or the other way around. Nevertheless, both factors, psychosocial and biological, appear to influence each other and seem to be part of a vicious circle leading to persisting MSK pain. Thus, attention should be paid to depressive feelings, pain beliefs, cognition, and behaviors when treating MSK pain.

### 4.1. Strengths and limitations

This study has several strengths, namely, the large sample size with the prospective population-based design and availability of information on several factors studied within the same study sample. Because of the study design, we could include data from questionnaires at different time points, including the assessment of history of MSK pain. Presence of MSK pain was also assessed at 6 year olds and not retrospectively, increasing the reliability of data and diminishing the risk on recall bias. Another strength is that at age 13 years, in the Generation R study, children themselves filled out the questionnaires, thus providing reliable data directly from the target population. The questionnaires for children were adjusted to be as understandable for their age and make their answers as objectively as possible.^25^ However, the reliability of the MSK pain data at the age of 6 years could be debated because this questionnaire was reported by parents, and young children may have difficulty describing the presence and nature of pain to their parents, and the parents may have difficulties with objectively reporting the nature of the pain.

There are some other limitations present in this study. First, as known in the general follow-up of the Generation R Study,^20^ selection bias toward a more healthy and Western population and higher socioeconomic status (SES) might have occurred and seen in the nonresponse analysis of the selected study sample. As a result of this selection, there may be an underestimation of pain because those of lower SES are more likely to report pain.

Moreover, because there were selective (ie, not random) missing values in the multivariable analysis, imputation was not possible. Nevertheless, univariate ORs in the total study sample (n = 3062) were similar (similar size and direction) but had smaller confidence intervals compared with the univariate ORs in the small study sample (n = 1612) in which the multivariable analyses were performed. Finally, because of the cross-sectional approach of the analyses, no inferences could be made on causality. Results should be interpreted with caution because multiple factors were tested without correction for multiple testing.

## 5. Conclusion

This study shows that MSK pain is already a frequent problem in 13-year-old children that often occurs daily and frequently has a chronic character. Moreover, female sex, lower maternal education, a higher BMI, being physically active, and behavioral problems were associated with an increased presence of MSK pain. To assess the causation of the associations and gain insight into underlying mechanisms, further research is needed. Especially longitudinal analyses into associations between different physical and psychosocial factors, particularly physical activity, BMI and behavioral problems, and the presence of MSK pain. These analyses could possibly lead to early interventions strategies and targeted approaches to address risk factors in children with MSK pain.

## Conflict of interest statement

S.B.-Z. reports grants from the Dutch Arthritis Association, the European Commission, ZonMw and reports personal fees from and consulting for Pfizer Infirst Healthcare outside of the submitted work. The remaining authors have no conflicts of interest to declare.

## Appendix A. Supplemental digital content

Supplemental digital content associated with this article can be found online at http://links.lww.com/PAIN/B999.

## Supplementary Material

SUPPLEMENTARY MATERIAL

## References

[1] Aartun E Hartvigsen J Wedderkopp N Hestbaek L. Spinal pain in adolescents: prevalence, incidence, and course: a school-based two-year prospective cohort study in 1,300 Danes aged 11-13. BMC Musculoskelet Disord 2014;15:187. 24885549 10.1186/1471-2474-15-187 PMC4045997

[2] Achenbach TM. Child behavior checklist. In: Kreutzer JS DeLuca J Caplan B, editors. Encyclopedia of clinical neuropsychology. New York, NY: Springer New York, 2011. p. 546–52.

[3] Briggs AM Cross MJ Hoy DG Sànchez-Riera L Blyth FM Woolf AD March L. Musculoskeletal health conditions represent a global threat to healthy aging: a report for the 2015 world health organization world report on ageing and health. Gerontologist 2016;56(suppl 2):S243–55. 26994264 10.1093/geront/gnw002

[4] Brouwer GM van Tol AW Bergink AP Belo JN Bernsen RMD Reijman M Pols HAP Bierma-Zeinstra SMA. Association between valgus and varus alignment and the development and progression of radiographic osteoarthritis of the knee. Arthritis Rheum 2007;56:1204–11. 17393449 10.1002/art.22515

[5] Cole TJ Lobstein T. Extended international (IOTF) body mass index cut-offs for thinness, overweight and obesity. Pediatr Obes 2012;7:284–94. 22715120 10.1111/j.2047-6310.2012.00064.x

[6] Costello EJ Copeland W Angold A. Trends in psychopathology across the adolescent years: what changes when children become adolescents, and when adolescents become adults? J Child Psychol Psychiatry 2011;52:1015–25. 21815892 10.1111/j.1469-7610.2011.02446.x PMC3204367

[7] Crossley KM Stefanik JJ Selfe J Collins NJ Davis IS Powers CM McConnell J Vicenzino B Bazett-Jones DM Esculier JF Morrissey D Callaghan MJ. 2016 Patellofemoral pain consensus statement from the 4th International Patellofemoral Pain Research Retreat, Manchester. Part 1: terminology, definitions, clinical examination, natural history, patellofemoral osteoarthritis and patient-reported outcome measures. Br J Sports Med 2016;50:839–43. 27343241 10.1136/bjsports-2016-096384 PMC4975817

[8] Dissing KB Hestbæk L Hartvigsen J Williams C Kamper S Boyle E Wedderkopp N. Spinal pain in Danish school children—how often and how long? The CHAMPS Study-DK. BMC Musculoskelet Disord 2017;18:67. 28343450 10.1186/s12891-017-1424-5 PMC5367004

[9] Felson DT Goggins J Niu J Zhang Y Hunter DJ. The effect of body weight on progression of knee osteoarthritis is dependent on alignment. Arthritis Rheum 2004;50:3904–9. 15593215 10.1002/art.20726

[10] Franz C Møller NC Korsholm L Jespersen E Hebert JJ Wedderkopp N. Physical activity is prospectively associated with spinal pain in children (CHAMPS Study-DK). Scientific Rep 2017;7:11598. 10.1038/s41598-017-11762-4 PMC5599496 28912463

[11] Fredriks AM van Buuren S Burgmeijer RJ Meulmeester JF Beuker RJ Brugman E Roede MJ Verloove-Vanhorick SP Wit JM. Continuing positive secular growth change in The Netherlands 1955-1997. Pediatr Res 2000;47:316–23. 10709729 10.1203/00006450-200003000-00006

[12] GBD 2017 DALYs and HALE Collaborators. Global, regional, and national disability-adjusted life-years (DALYs) for 359 diseases and injuries and healthy life expectancy (HALE) for 195 countries and territories, 1990-2017: a systematic analysis for the Global Burden of Disease Study 2017. Lancet 2018;392:1859–922. 30415748 10.1016/S0140-6736(18)32335-3 PMC6252083

[13] Groenewald CB Essner BS Wright D Fesinmeyer MD Palermo TM. The economic costs of chronic pain among a cohort of treatment-seeking adolescents in the United States. J Pain 2014;15:925–33. 24953887 10.1016/j.jpain.2014.06.002 PMC4150826

[14] Haraldstad K Sørum R Eide H Natvig GK Helseth S. Pain in children and adolescents: prevalence, impact on daily life, and parents' perception, a school survey. Scand J Caring Sci 2011;25:27–36. 20409061 10.1111/j.1471-6712.2010.00785.x

[15] Hawkins D Metheny J. Overuse injuries in youth sports: biomechanical considerations. Med Sci Sports Exerc 2001;33:1701–7. 11581555 10.1097/00005768-200110000-00014

[16] Heikkala E Paananen M Taimela S Auvinen J Karppinen J. Associations of co-occurring psychosocial and lifestyle factors with multisite musculoskeletal pain during late adolescence-A birth cohort study. Eur J Pain 2019;23:1486–96. 31070823 10.1002/ejp.1414

[17] Henschke N Harrison C McKay D Broderick C Latimer J Britt H Maher CG. Musculoskeletal conditions in children and adolescents managed in Australian primary care. BMC Musculoskelet Disord 2014;15:164. 24885231 10.1186/1471-2474-15-164 PMC4032455

[18] Huguet A Tougas ME Hayden J McGrath PJ Stinson JN Chambers CT. Systematic review with meta-analysis of childhood and adolescent risk and prognostic factors for musculoskeletal pain. PAIN 2016;157:2640–56. 27525834 10.1097/j.pain.0000000000000685

[19] Ivanova MY Achenbach TM Rescorla LA Harder VS Ang RP Bilenberg N Bjarnadottir G Capron C De Pauw SS Dias P Dobrean A Doepfner M Duyme M Eapen V Erol N Esmaeili EM Ezpeleta L Frigerio A Gonçalves MM Gudmundsson HS Jeng SF Jetishi P Jusiene R Kim YA Kristensen S Lecannelier F Leung PW Liu J Montirosso R Oh KJ Plueck J Pomalima R Shahini M Silva JR Simsek Z Sourander A Valverde J Van Leeuwen KG Woo BS Wu YT Zubrick SR Verhulst FC. Preschool psychopathology reported by parents in 23 societies: testing the seven-syndrome model of the child behavior checklist for ages 1.5-5. J Am Acad Child Adolesc Psychiatry 2010;49:1215–24. 21093771 10.1016/j.jaac.2010.08.019 PMC4247330

[20] Jaddoe V Duijn C Franco O Heijden A Iizendoorn M Jongste J Lugt A Mackenbach J Moll H Raat H Rivadeneira F Steegers E Tiemeier H Uitterlinden A Verhulst F Hofman A. The generation R study: design and cohort update 2012. Eur J Epidemiol 2012;27:739–56. 23086283 10.1007/s10654-012-9735-1

[21] Jones GT Macfarlane GJ. Epidemiology of low back pain in children and adolescents. Arch Dis Child 2005;90:312–6. 15723927 10.1136/adc.2004.056812 PMC1720304

[22] Kamper SJ Henschke N Hestbaek L Dunn KM Williams CM. Musculoskeletal pain in children and adolescents. Braz J Phys Ther 2016;20:275–84. 27437719 10.1590/bjpt-rbf.2014.0149 PMC4946844

[23] Kastelein M Luijsterburg PAJ Heintjes EM van Middelkoop M Verhaar JAN Koes BW Bierma-Zeinstra SMA. The 6-year trajectory of non-traumatic knee symptoms (including patellofemoral pain) in adolescents and young adults in general practice: a study of clinical predictors. Br J Sports Med 2015;49:400–5. 25431450 10.1136/bjsports-2014-093557

[24] King S Chambers CT Huguet A MacNevin RC McGrath PJ Parker L MacDonald AJ. The epidemiology of chronic pain in children and adolescents revisited: a systematic review. PAIN 2011;152:2729–38. 22078064 10.1016/j.pain.2011.07.016

[25] Kooijman MN Kruithof CJ van Duijn CM Duijts L Franco OH van IJzendoorn MH de Jongste JC Klaver CC van der Lugt A Mackenbach JP Moll HA Peeters RP Raat H Rings EH Rivadeneira F van der Schroeff MP Steegers EA Tiemeier H Uitterlinden AG Verhulst FC Wolvius E Felix JF Jaddoe VW. The Generation R Study: design and cohort update 2017. Eur J Epidemiol 2016;31:1243–64. 28070760 10.1007/s10654-016-0224-9 PMC5233749

[26] Krause BL Williams JP Catterall A. Natural history of Osgood-Schlatter disease. J Pediatr Orthop 1990;10:65–8. 2298897

[27] Krul M van der Wouden JC Schellevis FG van Suijlekom-Smit LW Koes BW. Musculoskeletal problems in overweight and obese children. Ann Fam Med 2009;7:352–6. 19597173 10.1370/afm.1005 PMC2713163

[28] Marshall WA Tanner JM. Variations in pattern of pubertal changes in girls. Arch Dis Child 1969;44:291–303. 5785179 10.1136/adc.44.235.291 PMC2020314

[29] Marshall WA Tanner JM. Variations in the pattern of pubertal changes in boys. Arch Dis Child 1970;45:13–23. 5440182 10.1136/adc.45.239.13 PMC2020414

[30] Nahit ES Hunt IM Lunt M Dunn G Silman AJ Macfarlane GJ. Effects of psychosocial and individual psychological factors on the onset of musculoskeletal pain: common and site-specific effects. Ann Rheum Dis 2003;62:755–60. 12860731 10.1136/ard.62.8.755 PMC1754627

[31] Paulis WD Silva S Koes BW van Middelkoop M. Overweight and obesity are associated with musculoskeletal complaints as early as childhood: a systematic review. Obes Rev 2014;15:52–67. 23941399 10.1111/obr.12067

[32] Perquin CW Hazebroek-Kampschreur A Hunfeld JAM Bohnen AM van Suijlekom-Smit LWA Passchier J van der Wouden JC. Pain in children and adolescents: a common experience. PAIN 2000;87:51–8. 10863045 10.1016/S0304-3959(00)00269-4

[33] Peterson H. Growing pains. Pediatr Clin North Am 1986;33:1365–72. 3786003 10.1016/s0031-3955(16)36147-8

[34] Picavet HSJ Berentzen N Scheuer N Ostelo R Brunekreef B Smit HA Wijga A. Musculoskeletal complaints while growing up from age 11 to age 14: the PIAMA birth cohort study. PAIN 2016;157:2826–33. 27780179 10.1097/j.pain.0000000000000724

[35] Quartana PJ Campbell CM Edwards RR. Pain catastrophizing: a critical review. Expert Rev Neurother 2009;9:745–58. 19402782 10.1586/ERN.09.34 PMC2696024

[36] Rathleff MS Roos EM Olesen JL Rasmussen S. High prevalence of daily and multi-site pain—a cross-sectional population-based study among 3000 Danish adolescents. BMC Pediatr 2013;13:191. 24252440 10.1186/1471-2431-13-191 PMC3840664

[37] Rathleff MS Skuldbøl SK Rasch MNB Roos EM Rasmussen S Olesen JL. Care-seeking behaviour of adolescents with knee pain: a population-based study among 504 adolescents. BMC Musculoskelet Disord 2013;14:225. 23899043 10.1186/1471-2474-14-225 PMC3729825

[38] Roth-Isigkeit A Thyen U Stöven H Schwarzenberger J Schmucker P. Pain among children and adolescents: restrictions in daily living and triggering factors. Pediatrics 2005;115:e152–62. 15687423 10.1542/peds.2004-0682

[39] Schellevis FG Westert G de Bakker DH Groenewegen P van der Zee J Bensing J. De tweede Nationale Studie naar ziekten en verrichtingen in de huisartsenpraktijk: aanleiding en methoden. Huisarts en Wetenschap 2003;46:956–62.

[40] Sebbag E Felten R Sagez F Sibilia J Devilliers H Arnaud L. The world-wide burden of musculoskeletal diseases: a systematic analysis of the World Health Organization Burden of Diseases Database. Ann Rheum Dis 2019;78:844–8. 30987966 10.1136/annrheumdis-2019-215142

[41] Sitthipornvorakul E Janwantanakul P Purepong N Pensri P van der Beek AJ. The association between physical activity and neck and low back pain: a systematic review. Eur Spine J 2011;20:677–89. 21113635 10.1007/s00586-010-1630-4 PMC3082686

[42] Stroud MW Thorn BE Jensen MP Boothby JL. The relation between pain beliefs, negative thoughts, and psychosocial functioning in chronic pain patients. PAIN 2000;84:347–52. 10666540 10.1016/s0304-3959(99)00226-2

[43] Sundblad GMB Saartok T Engström L-MT. Prevalence and co‐occurrence of self‐rated pain and perceived health in school‐children: age and gender differences. Eur J Pain 2007;11:171–80. 16542860 10.1016/j.ejpain.2006.02.006

[44] Tick NT van der Ende J Verhulst FC. Twenty-year trends in emotional and behavioral problems in Dutch children in a changing society. Acta Psychiatr Scand 2007;116:473–82. 17997726 10.1111/j.1600-0447.2007.01068.x

[45] van den Heuvel MM Jansen PW Bindels PJE Bierma-Zeinstra SMA van Middelkoop M. Musculoskeletal pain in 6-year-old children: the generation R study. PAIN 2020;161:1278–85. 31917774 10.1097/j.pain.0000000000001797

[46] van den Hoven LH Gorter KJ Picavet HS. Measuring musculoskeletal pain by questionnaires: the manikin versus written questions. Eur J Pain 2010;14:335–8. 19699125 10.1016/j.ejpain.2009.06.002

[47] Vargas-Prada S Coggon D. Psychological and psychosocial determinants of musculoskeletal pain and associated disability. Best Pract Res Clin Rheumatol 2015;29:374–90. 26612236 10.1016/j.berh.2015.03.003 PMC4668591

[48] Weiss JE Stinson JN. Pediatric pain syndromes and noninflammatory musculoskeletal pain. Pediatr Clin North Am 2018;65:801–26. 30031499 10.1016/j.pcl.2018.04.004

[49] Yang S Chang MC. Chronic pain: structural and functional changes in brain structures and associated negative affective states. Int J Mol Sci 2019;20:3130. 31248061 10.3390/ijms20133130 PMC6650904

