# Supplementary material for: Musculoskeletal pain in 13-year-old children: the generation R study
Source: Pain. 2024 Feb 7;165(8):1806–13. doi: 10.1097/j.pain.0000000000003182 (PMC11247448; doi:10.1097/j.pain.0000000000003182)
Supplement: SUPPLEMENTARY MATERIAL [file jop-165-1806-s001.pdf]

Supplementary Figure 1 – Pain mannequin with 61 possible locations and their MSK pain groups

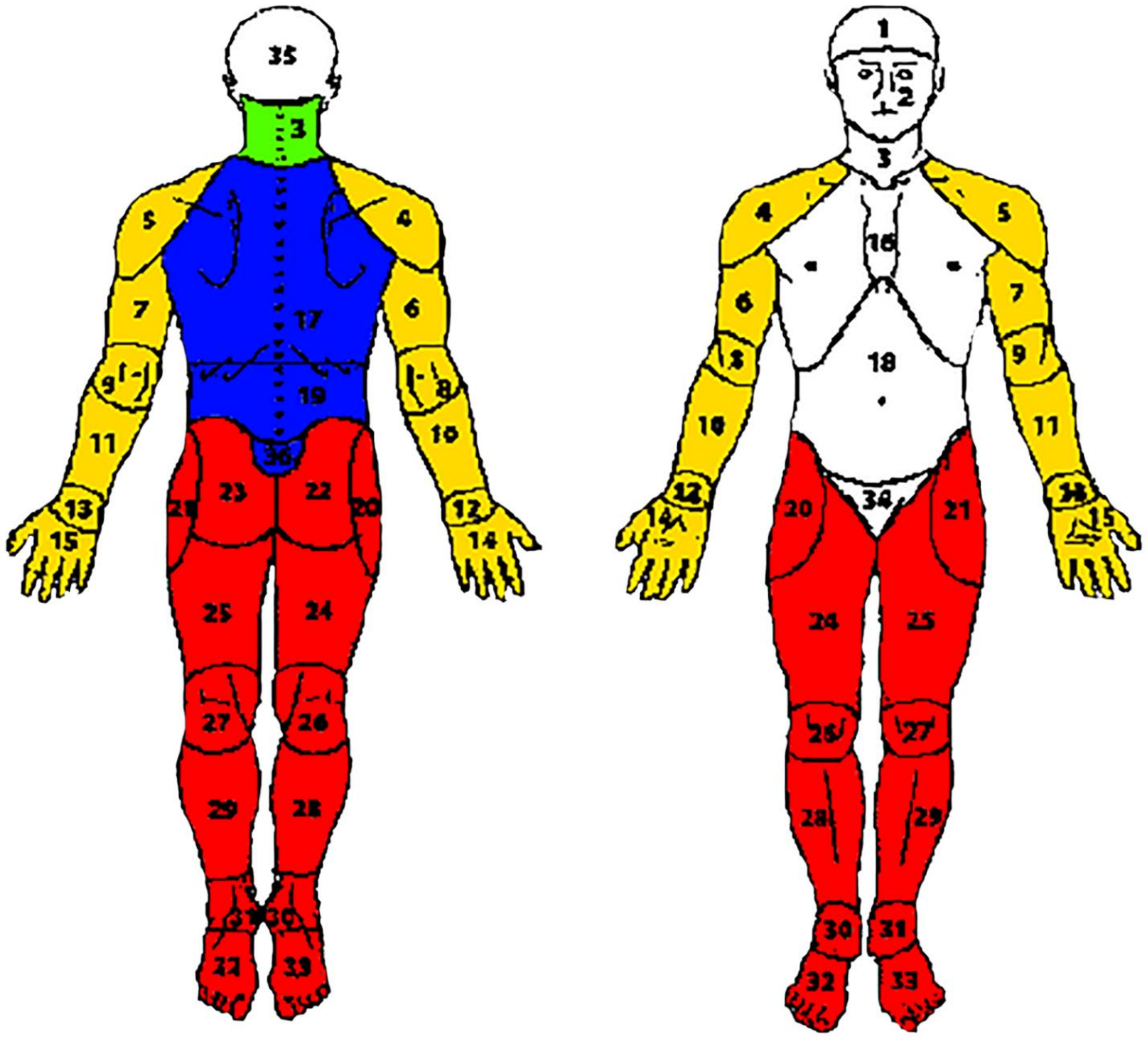

MSK pain: Neck (green), back (blue), upper limbs (yellow), Lower limbs (red)
Other (non-MSK pain): White

Supplementary Table 1 – Non-response analysis for the study sample

	**Included subjects (n=3062)**	**Excluded subjects (n=3779)**	***P***
• Sex			**0.001**
○ Girl	1594 (52.1%)	1808 (47.8%)	
○ Boy	1468 (47.9)	1971 (52.2)	
• Age, y	13.81 (13.58-14.36)	13.63 (13.46-14.00)	**0.001**
• Ethnicity			
○ Dutch	1853 (61.8)	2133 (58.6)	**0.006**
○ Other Western	270 (9.0)	307 (8.4)	
○ Non-Western	877 (29.2)	1197 (32.9)	
• Maternal educational level			
○ High	903 (30.3)	948 (26.2)	**0.001**
○ Intermediate	821 (27.5)	877 (24.3)	
○ Low	1260 (42.2)	1787 (49.5)	
• BMI, SD score	0.44 (1.19)	0.49 (1.21)	0.136

Values presented as number (%) for categorical factors, or median (interquartile range) or mean (SD) for continuous factors. This table is based on non-imputed data. Missings were 0 for gender, 1606 (23.5%) for age, 204 (3.0%) for ethnicity, 245 (3.6%) for maternal educational level and 1923 (28.1%) for BMI.
